# Modeling functional changes to *Escherichia coli* thymidylate synthase upon single residue replacements: a structure-based approach

**DOI:** 10.7717/peerj.721

**Published:** 2015-01-08

**Authors:** Majid Masso

**Affiliations:** Laboratory for Structural Bioinformatics, School of Systems Biology, George Mason University, Manassas, VA, USA

**Keywords:** Computational mutagenesis, Knowledge-based potential, Variant function prediction, Structure–function relationships, Machine learning, Thymidylate synthase

## Abstract

*Escherichia coli* thymidylate synthase (TS) is an enzyme that is indispensable to DNA synthesis and cell division, as it provides the only *de novo* source of dTMP by catalyzing the reductive methylation of dUMP, thus making it a key target for chemotherapeutic agents. High resolution X-ray crystallographic structures are available for TS and, owing to its relatively small size, successful experimental mutagenesis studies have been conducted on the enzyme. In this study, an *in silico* mutagenesis technique is used to investigate the effects of single amino acid substitutions in TS on enzymatic activity, one that employs the TS protein structure as well as a knowledge-based, four-body statistical potential. For every single residue TS variant, this approach yields both a global structural perturbation score and a set of local environmental perturbation scores that characterize the mutated position as well as all structurally neighboring residues. Global scores for the TS variants are capable of uniquely characterizing groups of residue positions in the enzyme according to their physicochemical, functional, or structural properties. Additionally, these global scores elucidate a statistically significant structure–function relationship among a collection of 372 single residue TS variants whose activity levels have been experimentally determined. Predictive models of TS variant activity are subsequently trained on this dataset of experimental mutants, whose respective feature vectors encode information regarding the mutated position as well as its six nearest residue neighbors in the TS structure, including their environmental perturbation scores.

## Introduction

*Escherichia coli* thymidylate synthase (TS; EC 2.1.1.45) drives the sole biosynthetic pathway for production of 2’-deoxythymidine 5’-monophosphate (dTMP), by using the cofactor 5,10-methylenetetrahydrofolate as a carbon donor to catalyze the reductive methylation of 2’-deoxyuridine 5’-monophosphate (dUMP), accompanied by the release of dihydrofolate (Santi & Danenberg, 1984). Owing to this essential role of TS in DNA synthesis and cell division, coupled with the enzyme’s relatively high degree of sequence and structural “core” conservation across numerous species (including human) (Finer-Moore, Montfort & Stroud, 1990), structure-based drug design efforts have led to the discovery of TS inhibitors that are now key components in certain anticancer treatment regimens (Jarmula, 2010). The native TS protein is functionally active as a symmetric dimer of two identical 30–35 kDa subunits, each consisting of 264 amino acid residues, with the same six-stranded *β*-sheet from both subunits packing against one other to form the dimer interface (Carreras & Santi, 1995). Two deep active site cavities are present in the structurally obligate TS homodimer, whereby lining each site are critical residues donated by both subunits (Carreras & Santi, 1995).

**Figure 1:**
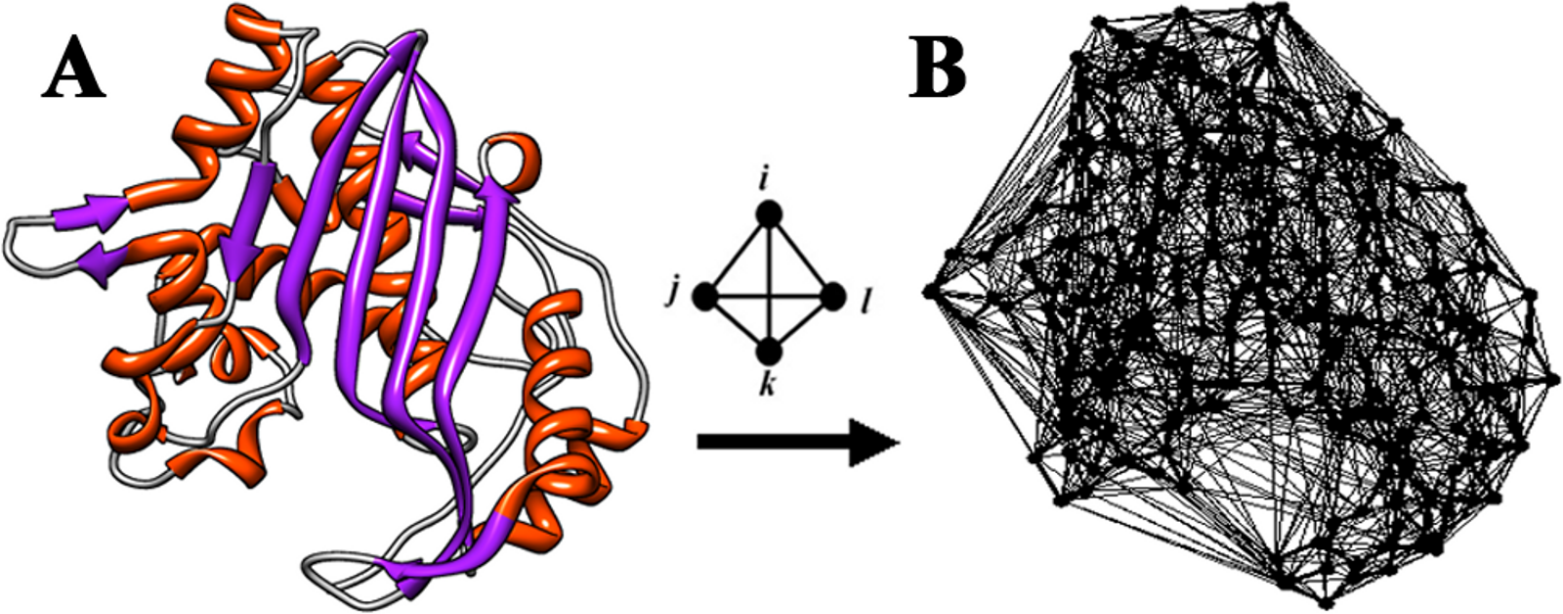
Delaunay tessellation of protein structure. (A) Ribbon diagram of the *E. coli* thymidylate synthase (TS) structure based on the Protein Data Bank (PDB) accession file 1f4b. (B) Delaunay tessellation of the TS structure coarse-grained at the amino acid level, with each residue represented by the coordinates of its constituent C-alpha atom in 3D space.

Included in the Protein Data Bank (PDB) (Berman et al., 2000) are X-ray crystallographic structures for both the monomeric TS polypeptide chain (Fig. 1A) and the biologically functional dimer (PDB accession codes 1f4b and 1kzi, respectively), each determined at 1.75 Å resolution (Erlanson et al., 2000; Fritz et al., 2002). Given the moderately small size of each TS subunit (1f4b consists of 263 amino acid residues, consecutively numbered 2–264), the protein is well suited for a variety of protein engineering experiments. In particular, site-directed mutagenesis studies of TS were previously undertaken via suppression of amber nonsense mutations, leading to the production of 372 variants of the enzyme generated by introducing the same subset of amino acids (A, C, E, F, G, H, K, L, P, Q, R, S, Y) at each of 30 targeted sequence positions, and yielding either 12 or 13 single residue replacements per position (Kim, Michaels & Miller, 1992; Michaels et al., 1990). These sites included completely substitutable exposed surface positions (E14, D105, N121, and E223), as well as positions well conserved across species that were substitutable to a surprisingly high degree (Q33, R35, D81, and R127) (Michaels et al., 1990). Another 12 sites accepted a limited number of substitutions, and these included residues that form parts of the substrate binding pockets (R21, W80, R126, H147, R166, D169, and N177), the active site nucleophile (C146), and important structural elements (F30, D110, Q151, and G204) (Michaels et al., 1990). Lastly, a subsequent study similarly investigated the impact of the single residue replacements at 10 sites forming parts of a surface loop (D20, T22, G23, and T24) that covers residues 20–24, as well as parts of a *β*-strand (G25, T26, L27, S28, I29, and G31) spanning residues 25–35; the latter contains a *β*-bulge centered over residues 30 and 31, while residues 30–35 occur at the dimer interface (Kim, Michaels & Miller, 1992). Residues surrounding the *β*-bulge, as well as three sites within the surface loop that are at the base of the substrate binding pocket, were found to be highly sensitive to amino acid substitutions (Kim, Michaels & Miller, 1992). The published experimental data on the qualitative activity levels of the TS variants, relative to that of the native TS, were used to categorize them as either unaffected (201 variants) or detrimentally affected (171 variants) by their respective residue replacements.

In this work, a structure-based *in silico* mutagenesis technique was implemented to quantitatively characterize every single residue TS variant (i.e., each of the 19 single amino acid replacements at every sequence position in the TS protein structure), one that relies on a knowledge-based four-body statistical potential energy function obtained by analyzing propensities of amino acid quadruplet interactions in over 1,400 diverse protein structures spanning the PDB. To generate the potential, each structure was initially coarse-grained at the residue level via the amino acid C-alpha atomic coordinates. For each protein, the set of C-alpha points were then all employed as vertices to create a space-filling 3-dimensional (3D) tetrahedral tiling of the structure, referred to as a Delaunay tessellation in the computational geometry literature (de Berg et al., 2008). Tessellation of an average-sized protein generates hundreds of packed tetrahedra, each objectively identifying at its four C-alpha vertices a quadruplet of nearest neighbor residues (Fig. 1B), and the four-body potential was derived using quadruplet frequency data obtained from these structures. Applications making use of this energy function mirror those common to traditional physics (i.e., molecular mechanics) based energy functions; in particular, as detailed in the Methods, the four-body potential is useful for calculating the total potential energy for any folded protein structure, as well as for computing structural residue environment scores for all the amino acids in the protein. These techniques were implemented here to model the native TS protein structure.

**Figure 2:**
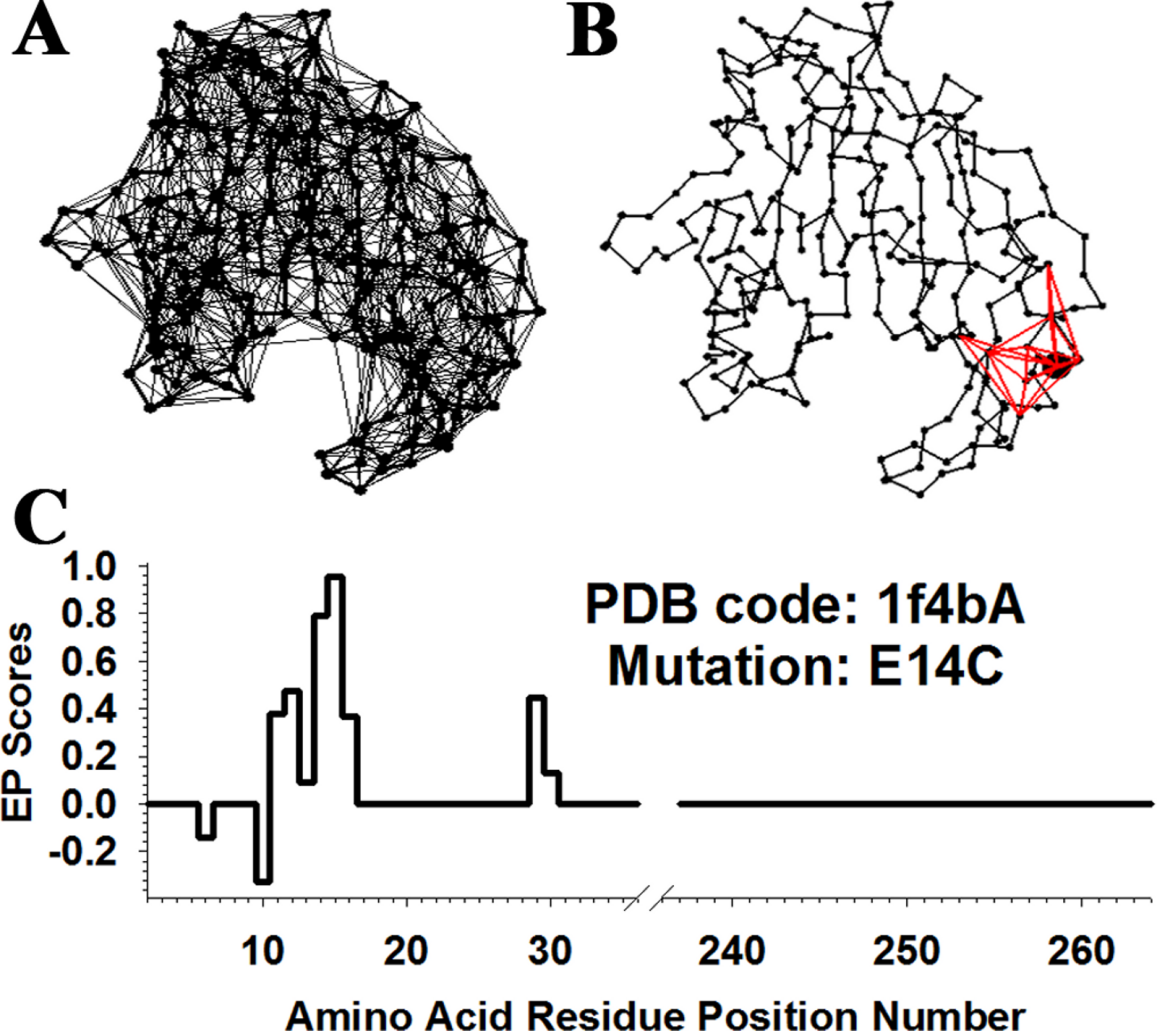
Visualization of the methodology. (A) Delaunay tessellation of *E. coli* thymidylate synthase (TS) from Fig. 1B, modified by the removal of tetrahedral edges longer than 12 Å to exclude false-positive residue quadruplet interactions from all subsequent analyses. (B) Ten tetrahedral simplices from the modified tessellation that all share as a vertex the C-alpha point representing residue E14, which is enlarged relative to the others. Collectively, there are nine additional C-alpha vertices associated with these simplices, and they represent TS residues forming the tessellation-based local structural neighborhood of E14. (C) Residual profile for the TS variant E14C. The ten residue positions with nonzero EP scores correspond precisely to the mutated position 14 and its nine neighbors, whose respective C-alphas collectively form the ten vertices of the simplices shown in (B). Attributes related to mutated position 14 and only its six closest neighbors, as determined by the lengths of simplex edges in (B), are included in the E14C variant feature vector.

Next, for each single residue substitution in the native TS enzyme, a computational mutagenesis approach employing the multibody potential described above was defined and used to empirically quantify structural environmental perturbation (EP) scores at the position undergoing the single residue mutation, as well as at all locally neighboring positions identified by tessellation of the 3D protein structure (Fig. 2). Consistent with the results of prior work analyzing protein-specific (Masso et al., 2014; Masso, Lu & Vaisman, 2006; Masso et al., 2009; Masso & Vaisman, 2007; Masso & Vaisman, 2013; Masso & Vaisman, 2011b) as well as collective (Masso & Vaisman, 2008; Masso & Vaisman, 2010; Masso & Vaisman, 2011a; Masso & Vaisman, 2014) sets of single residue mutants whose consequent functional changes had previously been experimentally determined, the structural EP scores corresponding to the 372 TS variants explored in this study were similarly capable of elucidating statistically significant structure–function relationships. Moreover, the EP scores were combined with additional sequence- and structure-based features (i.e., also referred to as predictors, input attributes, or independent variables with respect to computational modeling, as detailed in the Methods) in order to represent each TS variant as a 27D feature vector; and, when combined with the activity category of each TS variant (i.e., also referred to as the functional class, output attribute, or dependent variable, as detailed in the Methods), these data were used to train predictive models of TS variant activity by implementing four distinct cutting-edge statistical machine learning algorithms. In contrast to the previous studies, here the focus is on a highly conserved bacterial enzyme that served as an important target for the development of pharmaceutical inhibitor drugs. In particular, a “proof-of-principle” is reflected in this work via the successful analysis of yet another protein unrelated to any of those already investigated, a welcome outcome that could not be predetermined with any assurance. The results to follow establish that the TS structure is similarly capable of being modeled using the four-body statistical potential energy function, and that the TS variants can be accurately represented with the use of the related computational mutagenesis technique. Finally, the conceptual and analytical tools described and implemented in this work reflect a consolidation of methods previously developed and employed over the course of the earlier related studies. Of note, the experimental variant datasets of proteins previously analyzed using these computational techniques were generally larger and more uniformly distributed throughout their respective sequences relative to that for TS. Yet statistically significant observations and structure–function relationships made in those prior studies by applying these techniques are similarly reported here, reflecting a general robustness to the way in which variants are represented with this methodology.

## Methods

### Four-body potential derivation

High resolution X-ray crystallographic structures (<2.2 Å) for 1,417 diverse protein chains (<30% sequence identity), all having atomic coordinate data tabulated in PDB accession files (http://proteins.gmu.edu/automute/tessellatable1417.txt), were culled using the PISCES server (Wang & Dunbrack, 2003). The structures were coarse-grained at the amino acid level via the C-alpha atomic coordinates of the constituent residues, and the 3D point-set of each protein was then used to generate its Delaunay tessellation (de Berg et al., 2008), a tiled convex hull consisting of solid, space-filling, non-overlapping, irregular tetrahedra for which all C-alpha points participate as tetrahedral vertices (Fig. 1). Such a geometrical construction requires the four C-alpha vertices of every tetrahedron to be collectively closest to each other, thereby identifying in an objective way all quadruplets of nearest neighbor residues in the protein structure via tessellation. An adjacent pair of tetrahedra that border each other in the tessellation must share either one C-alpha vertex, one edge (i.e., two shared points), or one triangular facet (i.e., three shared points); furthermore, each C-alpha point is typically shared as a vertex by numerous adjacent tetrahedra in the packed 3D tiling, so the amino acid represented by that point simultaneously participates in several distinct nearest neighbor residue quadruplets (Fig. 2B) (Masso & Vaisman, 2010; Masso & Vaisman, 2014). To ensure that false-positive quadruplet interactions are eliminated from the tessellation, all tetrahedral edges longer than 12 Å (often between pairs of C-alphas that correspond to non-interacting distant residues on the surface, in order to complete the convex hull) are immediately removed prior to further analysis, effectively eliminating all tetrahedra that utilize those edges and revealing protein surface clefts and pockets via the tessellation (Fig. 2A) (Masso & Vaisman, 2008; Masso & Vaisman, 2010; Masso & Vaisman, 2014). All quantitative data associated with the Delaunay tessellations of protein structures were obtained by using the Qhull software package (http://www.qhull.org/) (Barber, Dobkin & Huhdanpaa, 1996); data formatting and analyses, both prior and subsequent to generating the tessellations, were performed using an ad-hoc suite of Perl codes written as needed; molecular graphics were produced with the UCSF Chimera package (Pettersen et al., 2004); and tessellation visualizations were generated using Matlab, Version 7.0.1.24704 (R14) Service Pack 1.

In this context, primary interest rests with detecting quadruplets of interacting residues via the four C-alpha vertices of every tetrahedron in these tessellations, irrespective of any particular order in which the four residues are written; hence, one arrangement type (e.g., CCDH, written in ascending alphabetical order) was singularly used as a representative for all possible permutations of the same four residues. Additionally, given that the sequences of protein structures contain multiple occurrences of the same amino acid types, a residue quadruplet identified at the four vertices of a tetrahedron may contain repeated instances of the same amino acids, as suggested by the above parenthetical example. By observing these constraints (i.e., all permutations of a tabulated quadruplet are excluded, and quadruplets may each contain repeated residues) and using a standard protein alphabet of *K* = 20 letters, the total number of distinct subsets of size *r* = 4 residues that can be specified is given by the combinatorial formula $\left({\scriptsize \begin{array}{c} \displaystyle K+r-1\\ \displaystyle r \end{array}}\right)=\left({\scriptsize \begin{array}{c} \displaystyle 23\\ \displaystyle 4 \end{array}}\right)=\text{8,855}$ (Masso & Vaisman, 2008; Masso & Vaisman, 2010; Masso & Vaisman, 2011a; Masso & Vaisman, 2014). For each such 4-residue subset (*i*, *j*, *k*, *l*), an observed relative frequency of occurrence *f_ijkl_* was calculated as the proportion of all tetrahedra generated by the 1,417 protein structure tessellations having the given quadruplet at its vertices, subsequent to removal of all edges longer than 12 Å. Next, by employing the multinomial probability distribution to obtain background (i.e., reference) frequencies, an expected rate of chance occurrence for each quadruplet was computed as ${p}_{i j k l}=\frac{4 !}{\prod _{n=1}^{20}\left({t}_{n}!\right)}\prod _{n=1}^{20}a_{n}^{{t}_{n}}$, where $\sum _{n=1}^{20}{a}_{n}=1$ and $\sum _{n=1}^{20}{t}_{n}=4$ (Masso & Vaisman, 2008; Masso & Vaisman, 2010; Masso & Vaisman, 2011a; Masso & Vaisman, 2014). Here, *a_n_* denotes the proportion of all residues comprising the 1,417 proteins that are of type *n*, and *t_n_* represents the number of repeated occurrences of residue type *n* in quadruplet (*i*, *j*, *k*, *l*). Based on a well-established application of the inverted Boltzmann principle, the log-likelihood score *s_ijkl_* = −log(*f_ijkl_*/*p_ijkl_*) is proportional to the (*i*, *j*, *k*, *l*) residue quadruplet multibody interaction energy (Sippl, 1993; Sippl, 1995); moreover, the combined set of scores for all 8,855 distinct quadruplet types defines the four-body statistical potential utilized in this study (http://proteins.gmu.edu/automute/potential_1417_cut12.txt) (Masso & Vaisman, 2008; Masso & Vaisman, 2010; Masso & Vaisman, 2014).

### Computational mutagenesis

For any tessellated protein structure (subject to the 12 Å edge-length cutoff), such as that of TS, the energy function derived above can be used for empirically calculating a *total potential* (*tp_wt_*) for the protein (i.e., total potential energy of the folded protein) as follows: first assign a score to each tetrahedron in the tessellation equal to the interaction energy of the residue quadruplet associated with its four C-alpha vertices, as tabulated in the above referenced four-body statistical potential, and then compute the sum of all these tetrahedral scores (Masso & Vaisman, 2007; Masso & Vaisman, 2010). A *residue environment score (RES)* can also be calculated for each primary sequence position number *i* in the protein structure, by adding together only scores of tetrahedra that share the C-alpha of that position as a vertex, where *q*_*i*,*wt*_ designates the RES value for each position of the native protein (Masso & Vaisman, 2008; Masso & Vaisman, 2010; Masso & Vaisman, 2014). Collectively, the vector $\lt {q}_{i,w t}{\mathop{\gt }\nolimits }_{i=1}^{n}$ (*n* = primary sequence length of protein structure) is referred to as a *3D-1D potential profile* (Bowie, Luthy & Eisenberg, 1991). Each RES value *q*_*i*,*wt*_ empirically provides an overall measure of how the residue at sequence position *i* interacts with all those at structurally nearby positions forming its local 3D neighborhood defined via tessellation (i.e., a measure of sequence-structure compatibility). The local structural neighbors of a given residue position consist of those whose C-alphas participate as vertices in the same tetrahedra as the C-alpha of that residue itself; more succinctly, the neighbors are precisely all those with C-alphas that are connected to the C-alpha of that residue position by a tetrahedral edge in the tessellation (Fig. 2B).

A single residue substitution is introduced at a protein sequence position in this scenario (i.e., in the tessellated protein structure) by associating the C-alpha vertex of that position with a new amino acid; hence, the tessellation construct itself is unaltered, and the modification involves changing a residue label at that point. This alters by one amino acid the residue quadruplets associated with all tetrahedra that share the vertex, thereby changing their tetrahedral scores. The RES values are also altered, say from *q*_*i*,*wt*_ to *q*_*i*,*mut*_, at the modified residue position itself and at all neighboring positions defined by the tessellation. At precisely these positions *i*, non-zero *environmental perturbation (EP)* scores are defined as *EP_i_* = *q*_*i*,*mut*_−*q*_*i*,*wt*_ and, given its significance in elucidating structure–function correlations, the term *residual score* is used in referring to the EP score at the mutated position (Masso & Vaisman, 2008; Masso & Vaisman, 2010; Masso & Vaisman, 2014). In particular, the residual score empirically quantifies relative change in global protein sequence–structure compatibility, as detailed in the next paragraph. Since *EP_i_* = 0 at all other positions *i* whose C-alpha vertices lie outside the structural neighborhood of the mutated position, this *in silico* mutagenesis technique clearly is also concerned with local residue effects. The vector $\lt E{P}_{i}~{\mathop{\gt }\nolimits }_{i=1}^{n}$ is termed the *residual profile* of the mutated protein (Fig. 2C) (Masso & Vaisman, 2008; Masso & Vaisman, 2010).

Next, the total potential of the mutated protein, denoted by *tp_mut_*, can be determined in the same way that *tp_wt_* was calculated for the native protein, by using the same tessellation modified by a single residue letter label alteration at the appropriate C-alpha vertex. It is a straightforward exercise to show that the difference *tp_mut_*−*tp_wt_* is precisely equivalent to the residual score (i.e., EP score at the mutated position) of the single residue variant (Masso & Vaisman, 2007; Masso & Vaisman, 2010); consequently, this computational mutagenesis models global structural effects of a mutation. Lastly, a *comprehensive mutational profile* (CMP_*i*_) score can be computed for each protein sequence position *i* by replacing the native residue with each of the 19 possible amino acid alternatives and averaging their respective residual scores (Masso, Lu & Vaisman, 2006). Thus, each CMP value quantifies the mean effect on protein sequence-structure compatibility by considering all possible substitutions of the native residue at the given position.

### Statistical learning and TS variant attributes

The Weka software package (http://www.cs.waikato.ac.nz/ml/weka/) (Frank et al., 2004; Witten & Frank, 2000) was used to implement four machine learning algorithms for this study: random forest (RF) (Breiman, 2001), support vector machine (SVM) (Platt, 1998), decision tree (DT) (Quinlan, 1993), and neural network (NN) (Witten & Frank, 2000). Relevant algorithm parameter values used for training were as follows: one hundred trees (i.e., iterations) for RF; fit logistic models to the outputs = true, complexity (C) = 2.0, epsilon = 10^−12^, standardized training data, and radial basis function (RBF) kernel with gamma = 0.01 for SVM; ten bagged (bootstrap aggregated) iterations and pruning confidence factor = 0.25 for DT; and two hidden layers, learning rate = 0.3, momentum = 0.2, and training time = 500 epochs for NN.

Despite their diverse methodological underpinnings, these supervised classification techniques all share the same goal of fitting a complex nonlinear function (i.e., model of the form *y* = *f*(*x*), where *x* and *y* are vectors) to data that distinctively characterize each of the 372 single residue TS variants with experimentally studied activity (i.e., the training set of known examples). Here, the single residue TS mutants were encoded as feature vectors sharing a common set of components (i.e., the input attributes or independent variables *x_i_*, *i* = 1, 2, …, *N* of the model). Values for the input attributes are variant-specific, providing a unique feature vector representation for each TS mutant, and the objective is to evaluate their usefulness as predictors of TS variant activity (i.e., categorical U/A output attributes or dependent variables *y_i_*, *i* = 1, 2 of the model).

In particular, the input attributes used for characterizing each single residue TS variant included the following (Masso & Vaisman, 2010; Masso & Vaisman, 2014): primary sequence position number of the mutated residue, identities of the native and replacement amino acid residues, and the residual score (i.e., the EP score at the mutated position). Based on the local structural neighborhood of the mutated position as defined by the tessellation of TS, additional feature vector components consisted of the EP scores at the six nearest neighbor positions, ordered by proximity to the mutated position (i.e., 3D Euclidean distance as measured by the length of tetrahedral edges between respective C-alpha pairs). The amino acid identities at the six nearest neighbors, and their sequence locations relative to the mutated position (i.e., difference between neighbor and mutated position primary sequence numbers), were also included in the feature vector and similarly ordered as the EP scores of the neighbors. Lastly, the following input attributes were added to each TS variant feature vector:

(1) Mean volume and mean tetrahedrality calculated for the subset of tetrahedra in the TS tessellation that share the mutated position as a vertex, where tetrahedrality is given by $\sum _{i\gt j}({l}_{i}-{l}_{j})^{2}/15{\bar {l}}^{2}$ such that *l_i_* measures the length of the *i*th edge of the tetrahedron and $\bar {l}$ is the mean length of all six tetrahedral edges;
(2) Secondary structure at the mutated position (H, helix; S, strand; or C, coil);
(3) Mutated position depth (S, surface; U, undersurface; or B, buried), a tessellation-based measure of surface accessibility. If the mutated position serves as a vertex of a triangular facet for precisely one tetrahedron (i.e., the facet is not shared by two adjacent tetrahedra), then the position is on the surface. An undersurface position is one connected to a surface position via a tetrahedral edge. All other positions are buried;
(4) The number of tessellation edges the mutated position shares with surface positions (zero by definition for buried positions).

Hence, a total of 27 input attributes were evaluated for each TS variant. An output attribute was also associated with each TS variant and defined to be the effect of the mutation on the level of activity, a categorical variable taking one of two possible values: unaffected (U) or detrimentally affected (A).

### Evaluating model performance

Leave-one-out cross-validation (LOOCV) as well as tenfold cross-validation (10-fold CV) testing procedures were implemented for evaluating the performance of models trained on the experimental dataset of 372 single residue TS variants with known effects on activity. As both approaches produced similar results, those based on LOOCV testing were reported in nearly all instances; an exception was made in the production of learning curves to visualize how training set size impacts performance, for which 10-fold CV testing data were used in creating the plots. To implement a 10-fold CV procedure in general, the training set instances (e.g., 372 TS variants with known activity) are randomly stratified to ten disjoint subsets roughly equal in size, and testing then proceeds as follows: one subset is held-out while a model is trained using all of the variants from the other nine subsets combined; the model is used to predict activity categories for variants in the held-out subset based on the values of the input attributes in their feature vectors; the process is iterated so that each subset serves once as a hold-out and has its variants predicted by the model trained using the combined variants from the other nine subsets; and overall performance is calculated based on the aggregate of correct predictions and misclassifications obtained for all 372 TS variants (Witten & Frank, 2000). Implementation of LOOCV proceeds in a similar fashion, except that the number of initial subsets is equivalent to the size of the training set (i.e., each subset is a singleton containing one TS variant). The results of any two independent runs of 10-fold CV often yield minor differences, due to variability in the way variants are randomly segregated initially to form ten disjoint subsets, so the overall results are reported as an average of those obtained by ten independent iterations of the procedure; in this regard, LOOCV is a deterministic method (i.e., identical results guaranteed with every run) requiring only a single iteration (Witten & Frank, 2000).

The performance of each testing procedure was determined by referring to the variant activity categories as Positive (P) and Negative (N), where P = class of unaffected (U) variants and N = class of detrimentally affected (A) variants; hence, TP and TN represent the total number of true (i.e., correct) predictions from within each category, while FN and FN correspond to the total number of respective misclassifications. Using this notation, predictions were evaluated by calculating sensitivity = TP / (TP + FN), specificity = TN / (TN + FP), and PPV = positive predictive value (i.e., precision) = TP / (TP + FP). Additionally, the following quantities were computed: balanced accuracy rate BAR = 0.5 × [Sensitivity + Specificity]; Matthew’s correlation coefficient

\begin{eqnarray*} \text{MCC}=\frac{\text{TP}\times \text{ TN}-\text{ FP}\times \text{ FN}}{\sqrt{(\text{TP}+\text{ FN)(TP}+\text{ FP)}(\text{TN}+\text{ FN)(TN}+\text{ FP)}}}; \end{eqnarray*}

and the area (AUC) under the receiver operating characteristic (ROC) curve, a plot of the true-positive rate (i.e., Sensitivity) versus false-positive rate (i.e., 1 − Specificity) in the unit square. The AUC is equivalent to a non-parametric Wilcoxon test of ranks (Hanley & McNeil, 1982), taking on values that fall within two extremes given by AUC ≈ 0.5 (random guessing) and AUC = 1.0 (perfect classifier).

## Results and Discussion

### *E. coli* TS structure–function relationships

**Figure 3:**
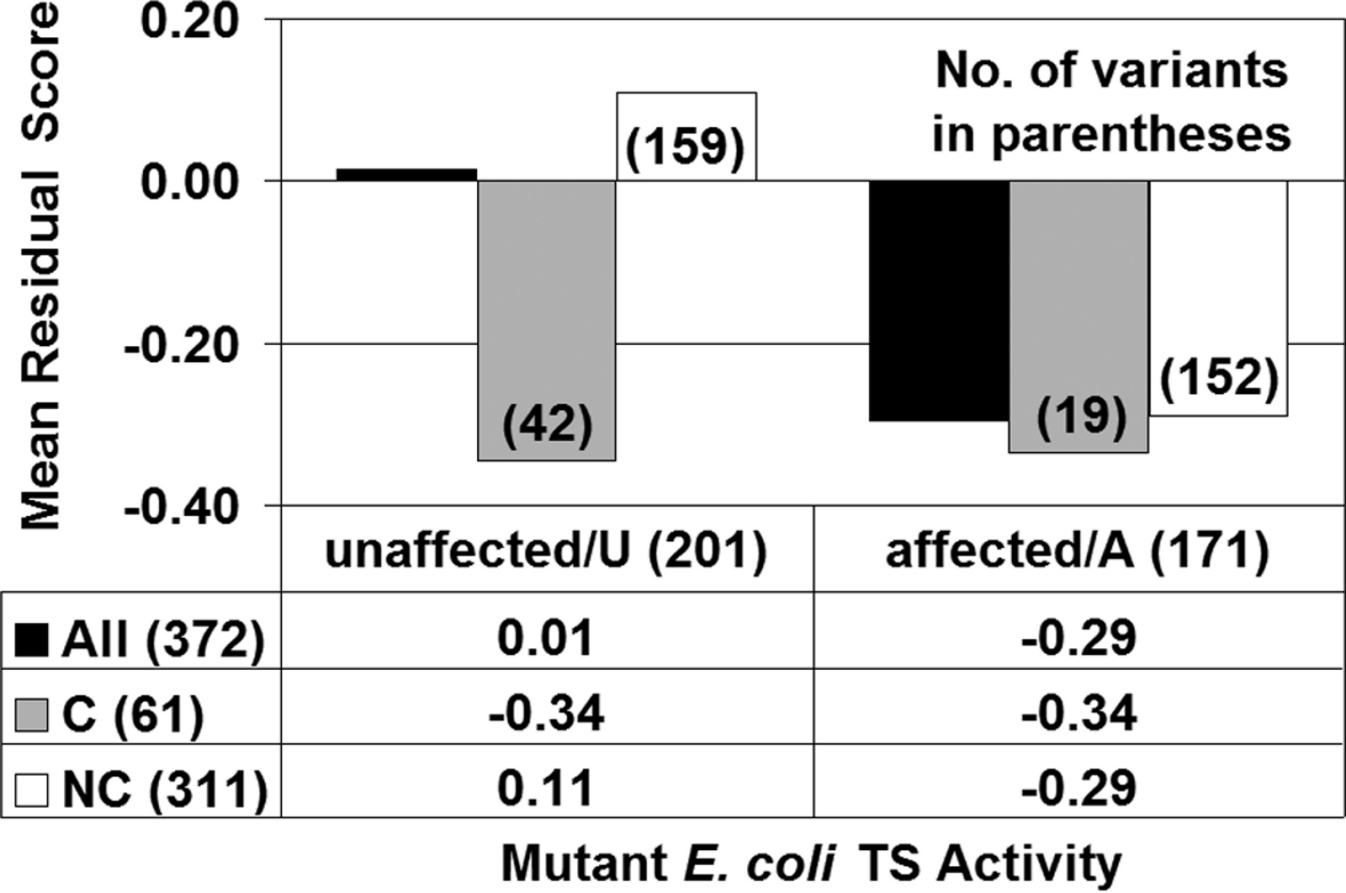
*E. coli* thymidylate synthase (TS) structure–function correlation. C/NC refer to conservative/non-conservative amino acid substitutions.

A residual profile was derived for each TS variant, categorized as either unaffected (U, 201 variants) or detrimentally affected (A, 171 variants) based on experimentally determined activity, by computing its EP scores at all sequence positions in the TS protein structure. Focusing specifically on the residual score of each TS variant (i.e., the EP score at the mutated position) and the calculated average of such scores over all variants comprising each activity class (i.e., categorical mean residual scores), Fig. 3 (row labeled All) reveals that TS protein functional impairment upon mutation is correlated with a detrimental impact to TS protein structure (i.e., mean residual score of activity class U is positive with relatively small magnitude, while that of class A is negative with substantially larger magnitude). Moreover, the difference between mean residual scores for the U/A activity class pair is statistically significant (*t*-test: *p* < 0.05).

Variants in each class were further categorized based on whether the replacement residue represented a conservative (C) or non-conservative (NC) substitution relative to the native amino acid, and mean residual scores were computed for each of these subgroups. By clustering amino acids into six groups as [(A, S, T, G, P), (D, E, N, Q), (R, K, H), (F, Y, W), (V, L, I, M), (C)] based on physicochemical similarities, intraclass residue replacements are defined as conservative while interclass substitutions are non-conservative (Dayhoff, Schwartz & Orcut, 1978). As depicted in Fig. 3, the non-conservative variant subsets within each activity category clearly drive the overall structure–function relationship; furthermore, the conservative variants within each activity category display a deleterious average effect on TS structure (i.e., mean residual scores are −0.34 for both C subsets in Fig. 3), contrary to an expectation that conservative substitutions would minimally impact structure in the aggregate (i.e., mean residual scores that are closer to zero). The latter observation stems from bias that exists among the 372 experimental TS variants for residue substitutions at highly intolerant positions, as opposed to uniform sampling from among all conservative TS variants, a fact supported by prior computational studies on proteins for which comprehensive experimental mutagenesis data were available for analysis (Masso et al., 2008; Masso, Lu & Vaisman, 2006; Masso et al., 2009; Masso & Vaisman, 2011b).

An alternative analysis was performed by examining the way in which these 372 experimental TS mutants were distributed throughout a 2 × 4 contingency table having activity categories and residual score intervals as row and column headings, respectively. In particular, the two U/A activity classes were used to label the table rows, while four clusters of residual scores formed by the intervals (−∞, −1), [−1, 0), [0, 1), and [1, + ∞) were used to identify the columns, and each cell in the table contained the number of TS variants satisfying the respective row and column conditions. A chi-square test applied to the table led to rejection of the null hypothesis that no association exists between activity level and residual scores (*χ*^2^ = 33.91, 3 degrees of freedom; *p* < 0.0001).

### Classification of *E. coli* TS residue positions

**Figure 4:**
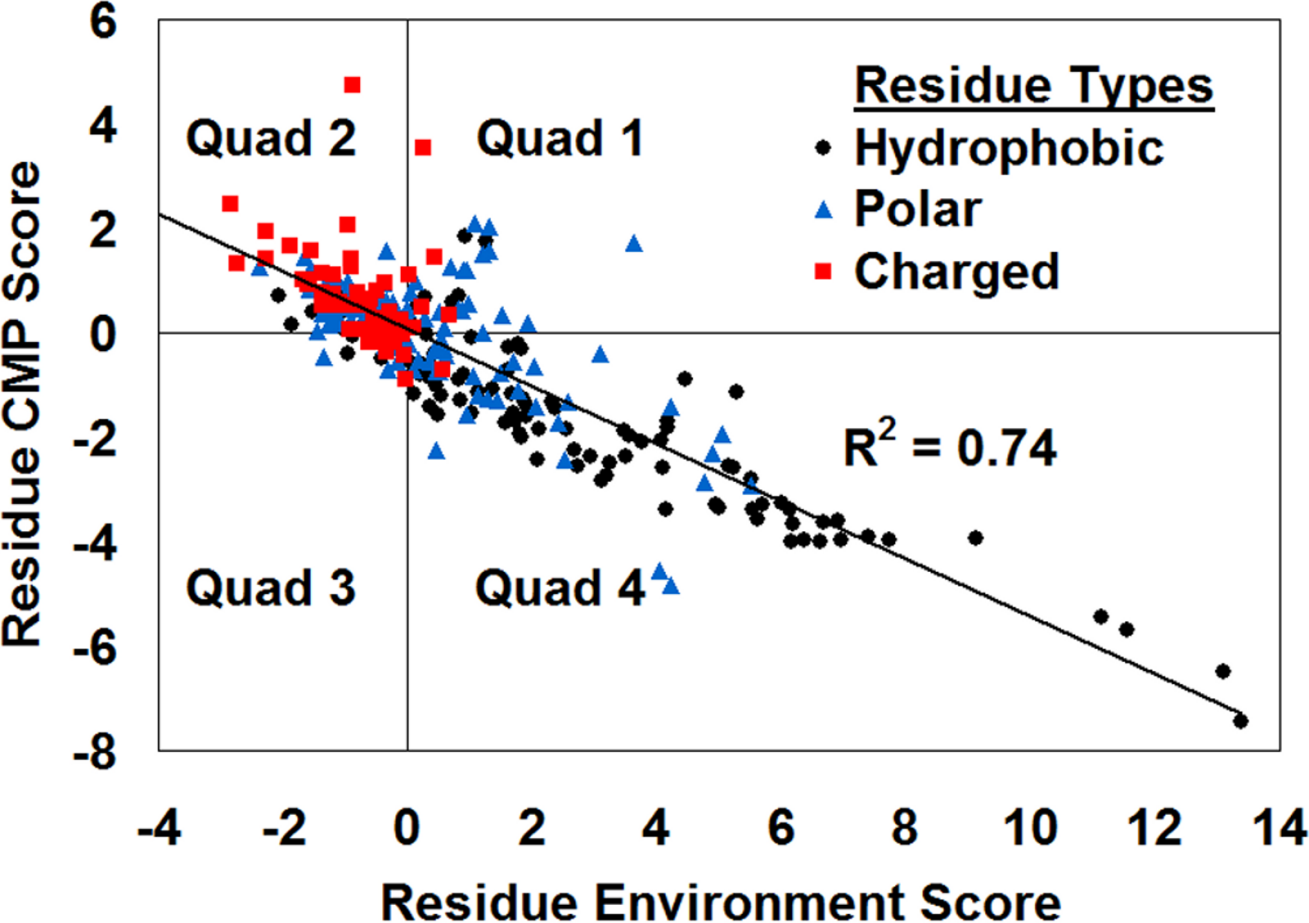
CMP—potential profile correlation plot for *E. coli* thymidylate synthase. Note how the amino acid residues comprising the protein are clustered by polarity.

A closer inspection of the *in silico* comprehensive single residue mutagenesis data and residue environment scores at all 263 constituent sequence positions in the TS protein structure (PDB accession code 1f4b) revealed a strong inverse correlation (*R*^2^ = 0.74) between CMP and RES scores (Fig. 4). When the residual scores of non-conservative (NC) and conservative (C) residue substitutions at each position were averaged separately, the resulting modified NC-CMP and C-CMP data showed NC substitutions (*R*^2^ = 0.74) to be the driving force behind the overall correlation in Fig. 4, with minimal contribution from C substitutions (*R*^2^ = 0.10). Similar results were repeatedly observed with the use of analogous *in silico* data obtained from a variety of diverse proteins, including HIV-1 protease (Masso, Lu & Vaisman, 2006; Masso & Vaisman, 2003), *E. coli lac* repressor (Masso et al., 2008), bacteriophage f1 gene V protein (Masso et al., 2009), bacteriophage T4 lysozyme (Masso, Alsheddi & Vaisman, 2009) and human interleukin-3 (Masso & Vaisman, 2011b), whereby an identical pattern of constituent amino acid residue clustering by polarity emerged in each instance (Fig. 4: hydrophobic/apolar, Quad 4; charged, Quad 2; polar, diffuse pattern about the origin). Moreover, application of a chi-square test to the 4 × 3 contingency table (Table 1) quantifying the distribution of all residues in the TS structure as depicted in Fig. 4, whereby Cartesian coordinate quadrant locations (Quads 1–4) and residue polarities (apolar, charged, polar) designated row and column headings, respectively, led to rejection of the null hypothesis that no association exists between polarity and location (*χ*^2^ = 103.32, 6 degrees of freedom; *p* < 0.0001).

**Table 1:** Distribution of all TS residues.

	Residue types			
Graph quads	Apolar	Charged	Polar	Total
Q1	8	6	21	35
Q2	11	44	38	93
Q3	7	8	9	24
Q4	77	1	33	111
Total	103	59	101	263

Next, a detailed analysis was performed using a subset of 107 annotated TS residues, taking into consideration structural locations and functional properties. In particular, 34 amino acids (L7, M8, V11, L38, F42, L59, F62, L72, V77, L90, V93, W98, I112, V115, L119, I128, V130, M141, F150, L159, L163, V170, F171, L174, L184, V185, M187, M188, F199, W201, L208, L230, I239, and F247) were determined to be buried by the GETAREA (http://curie.utmb.edu/getarea.html) program (Fraczkiewicz & Braun, 1998); 8 catalytic residues (E58, W80, Y94, C146, H147, R166, D169, and N177) were identified by accessing the Catalytic Site Atlas (http://www.ebi.ac.uk/thornton-srv/databases/CSA/) (Furnham et al., 2014) and by referring to Dev et al. (1989); 33 dimer interface residues (T16, K18, N19, D20, S28, F30, Q33, R35, W101, T103, P104, D124, R126, I129, S131, W133, V135, G136, A148, Q151, Y153, V154, A155, D156, S160, Q162, Y164, S167, V200, T202, D205, H207, and Y209) were reported in Greene et al. (1993); and 32 amino acids (K2, D13, E14, Q17, G23, D40, E74, N76, E86, N87, D105, G106, R107, N121, D122, D139, D193, D214, L218, S221, E223, P226, K233, K235, E237, E245, G251, D253, P256, K259, P261, and I264) were deemed exposed both by using the tessellation-based definition of depth as well as by applying the GETAREA program. Distribution of the residues belonging to each structural or functional subgroup according to their Cartesian coordinate quadrant locations, as depicted in Fig. 4, is summarized in Table 2. Fisher’s exact test applied to this 4 × 4 contingency table led to rejection of the null hypothesis that no association exists between structural/functional subgroups and quadrant locations (*p* < 0.0001).

**Table 2:** Distribution of annotated TS residues.

	Residue Types				
Graph quads	Buried^a^	Catalytic^b^	Exposed^c^	Interface^d^	Total
Q1	0	2	0	7	9
Q2	0	2	22	8	32
Q3	0	0	8	6	14
Q4	34	4	2	12	52
Total	34	8	32	33	107

**Notes.**

a GETAREA (http://curie.utmb.edu/getarea.html) using PDB file 1f4b (TS monomer).

b Catalytic Site Atlas (http://www.ebi.ac.uk/thornton-srv/databases/CSA/) using PDB file 1f4b, as well as Dev et al. (1989).

c Overlap between surface residues identified using both GETAREA, with PDB file 1kzi (TS dimer), and the tessellation-based definition of depth, excluding any residues annotated as either interface or catalytic.

d Greene et al. (1993).

**Figure 5:**
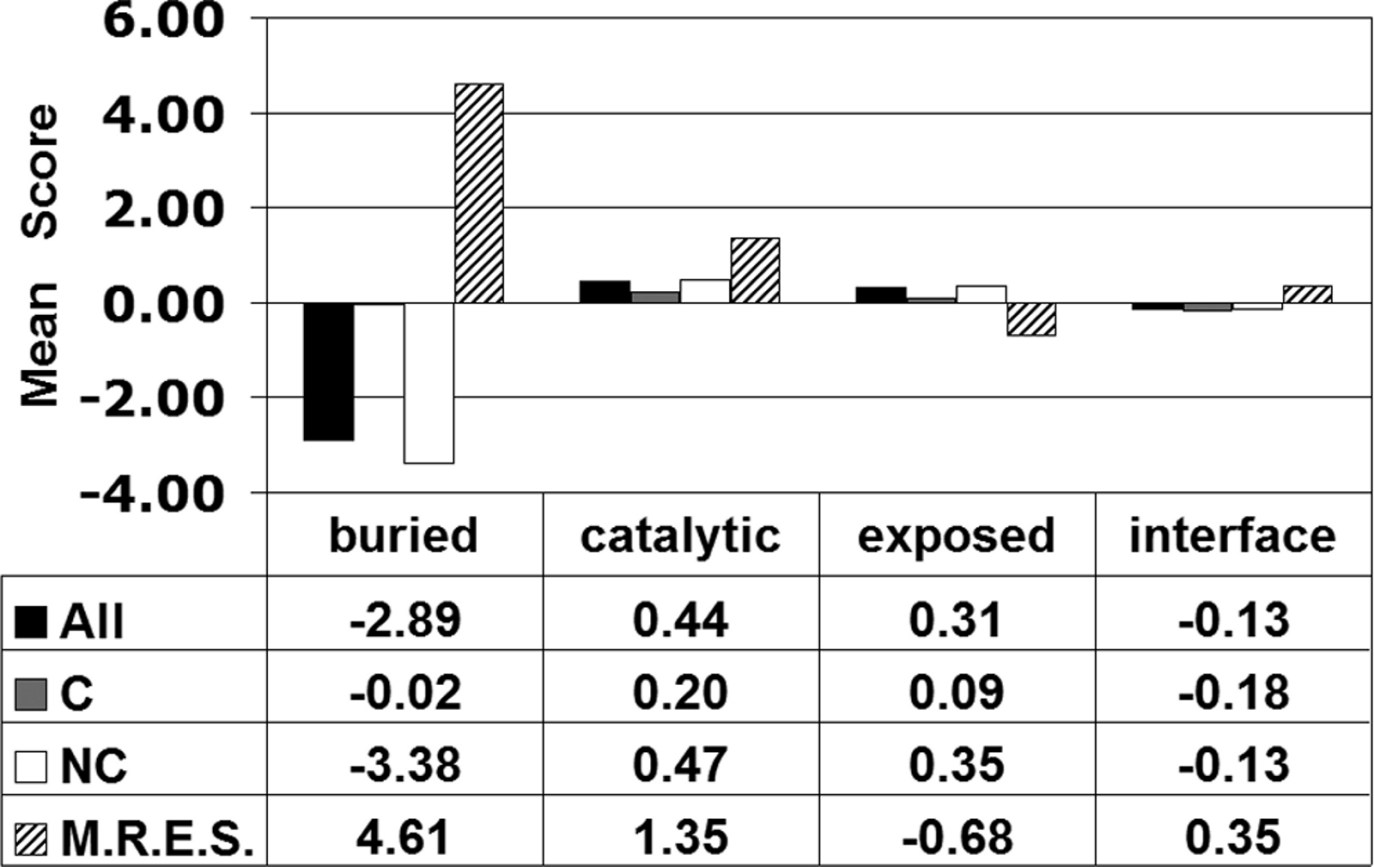
Characterization of *E. coli* thymidylate synthase structural/functional residue groups. C/NC refer to conservative/non-conservative amino acid substitutions, and M.R.E.S. refers to mean of the residue environment scores.

These annotated residue positions were subsequently characterized via their respective *in silico* data, where Fig. 5 depicts both the mean of the residue environment scores (M.R.E.S.) over all the positions of each subgroup, as well as the mean of the residual scores computed for all 19 single residue replacements at all positions within each subgroup (rows labeled All/C/NC). It is clear from Fig. 5 that these mean scores differ substantially between buried and exposed residues; furthermore, the scores distinguish interface residues from other exposed residues, while mean scores for the set of catalytic residues display a pattern that is distinct from those for the other three subgroups.

### Machine learning models for predicting *E. coli* TS variant activity

**Figure 6:**
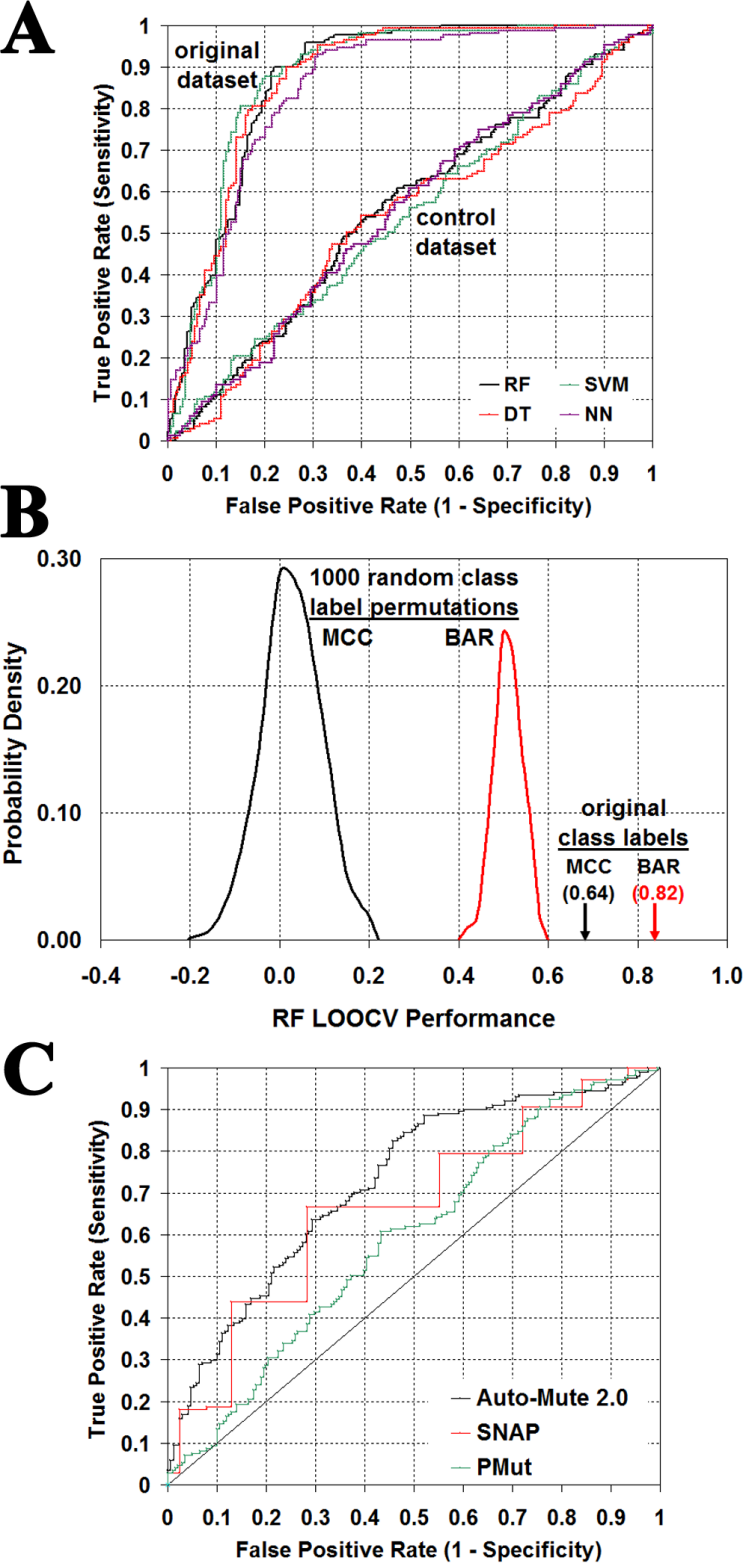
Statistical significance of classifier performance. (A) Leave-one-out cross-validation (LOOCV) ROC curves obtained for all four models based on the original dataset as well as a control generated by a single random shuffling of the U (unaffected)/A (detrimentally affected) activity class labels among the 372 *E. coli* thymidylate synthase (TS) variants in the dataset. (B) Distribution of LOOCV random forest (RF) prediction performance over 1,000 random activity class label permutations, compared with results using the original dataset (BAR, balanced accuracy rate; MCC, Matthew’s correlation coefficient). (C) Comparison of ROC curves corresponding to TS variant predictions obtained with three state-of-the-art methods.

Four supervised classification models were trained using the dataset of 372 experimental single residue TS variants with known activity (i.e., expressed as a U/A categorical output attribute), where each variant was uniquely represented as a 27D feature vector of input attributes consisting of EP scores, calculated using the *in silico* mutagenesis technique, as well as sequence- and structure-based data, derived from both the TS structure and its tessellation (see Methods for details). The trained models were derived by implementing the random forest (RF), support vector machine (SVM), decision tree (DT), and neural network (NN) machine learning algorithms. Models were evaluated based on the accuracy of predictions obtained via leave-one-out cross-validation (LOOCV) testing, as reported in the upper section of Table 3, whereby all four methods performed equally well and consistent with one another. In every case, the information encoded by the feature vector input attributes proved to be invaluable for accurately distinguishing between TS variants categorized by activity as either unaffected (U) or detrimentally affected (A). To highlight the significance of these signals with respect to all four trained models, LOOCV testing results in Table 3 using the original dataset were compared with those obtained using a control dataset generated by randomly shuffling the 201U/171A class labels among the 372 TS variants. Dramatic drops in AUC values to levels near 0.5 were observed using the control dataset (Fig. 6A), suggesting these model predictions were equivalent to random guessing, a conclusion further supported by BAR and MCC performance measures: RF (AUC = 0.55, BAR = 0.55, MCC = 0.10), SVM (AUC = 0.54, BAR = 0.53, MCC = 0.05), DT (AUC = 0.53, BAR = 0.56, MCC = 0.13), and NN (AUC = 0.55, BAR = 0.53, MCC = 0.07).

**Table 3:** Evaluation of TS variant prediction performance.

Method	Sensitivity	Specificity	PPV	MCC	BAR	AUC
**LOOCV testing results**						
RF	0.79	0.85	0.86	0.64	0.82	0.87
SVM	0.81	0.85	0.86	0.66	0.83	0.88
DT	0.77	0.87	0.88	0.64	0.82	0.87
NN	0.77	0.81	0.83	0.58	0.79	0.85
**Predictions made by existing methods**						
Auto-Mute 2.0	0.95	0.50	0.63	0.38	0.73	0.73
SNAP	0.32	0.99	0.98	0.40	0.65	0.67
PMut	0.27	0.87	0.71	0.17	0.57	0.59

For a more systematic approach to assessing statistical significance of the LOOCV results presented in Table 3, 1,000 control sets were generated as before via class label permutations (i.e., random class shuffles), and each dataset was used to train an RF model and evaluate performance measures via LOOCV testing. All calculated BAR and MCC values based on these controls were found to be distributed within narrow windows centered around 0.5 and zero (Fig. 6B: BAR = 0.50 ± 0.03, MCC = 0.00 ± 0.07), respectively, and distant from those obtained using the original arrangement of the class labels (Table 3: BAR = 0.82, MCC = 0.64), so the *p*-value for predictive power of the model is less than 0.001. Nearly identical LOOCV testing results were obtained when models based on the other three algorithms were trained using the control sets: SVM (BAR = 0.50 ± 0.04, MCC = 0.00 ± 0.08), DT (BAR = 0.50 ± 0.03, MCC = 0.00 ± 0.07), and NN (BAR = 0.50 ± 0.03, MCC = 0.00 ± 0.06). Comparing these data with LOOCV testing results in Table 3 obtained using the original dataset revealed the same degree of statistical significance in each of these cases as that observed with the RF algorithm.

Furthermore, these 372 TS variants were submitted to three existing state-of-the-art models in order to obtain predictions (lower section of Table 3, Fig. 6C): Auto-Mute 2.0 (http://proteins.gmu.edu/automute) (Masso & Vaisman, 2014), SNAP (https://www.rostlab.org/services/snap/) (Bromberg & Rost, 2007), and PMut (http://mmb2.pcb.ub.es:8080/PMut/) (Ferrer-Costa et al., 2005). The Auto-Mute 2.0 model was trained on 8,561 single residue mutations (5,251 U / 3,310 A) occurring in seven diverse proteins (Masso & Vaisman, 2011a), exclusive of TS, so that the TS variant data represent an independent test set. The same is true for PMut, which was trained using only mutations from human proteins (i.e., single nucleotide polymorphisms, or SNPs), although subsequent studies showed that this model could also be used to predict protein variants from other organisms. The SNAP model, however, was trained using the annotated variants listed in the Protein Mutant Database (PMD) (Kawabata, Ota & Nishikawa, 1999), among which these TS variants are all included; hence, SNAP has a significant advantage whereby the TS variant test set is not at all independent, and prediction performance in this case reflects the resubstitution error (i.e., how well a model fits data it has already seen and on which it was trained). Additionally, Auto-Mute 2.0 utilizes all but one of the input attributes applied in this study, the exception being the sequence position number of the mutated residue (i.e., the Auto-Mute 2.0 model is universal and not protein-specific), while SNAP and PMut both incorporate information derived from multiple sequence alignments. Given that the TS variant feature vectors used in both the present study as well as Auto-Mute 2.0 did not include input attributes based on such evolutionary information, the work here corresponds to an orthogonal approach that is complementary to the SNAP and PMut methods. Overall, Auto-Mute 2.0 predictions (Table 3, Fig. 6C) displayed considerably more balance and less skew toward one activity category, as evidenced by the calculated Sensitivity and Specificity values, leading to higher accuracy (BAR) and AUC measures and outperforming the other two methods.

### Characteristics of *E. coli* TS variant-specific predictions

**Figure 7:**
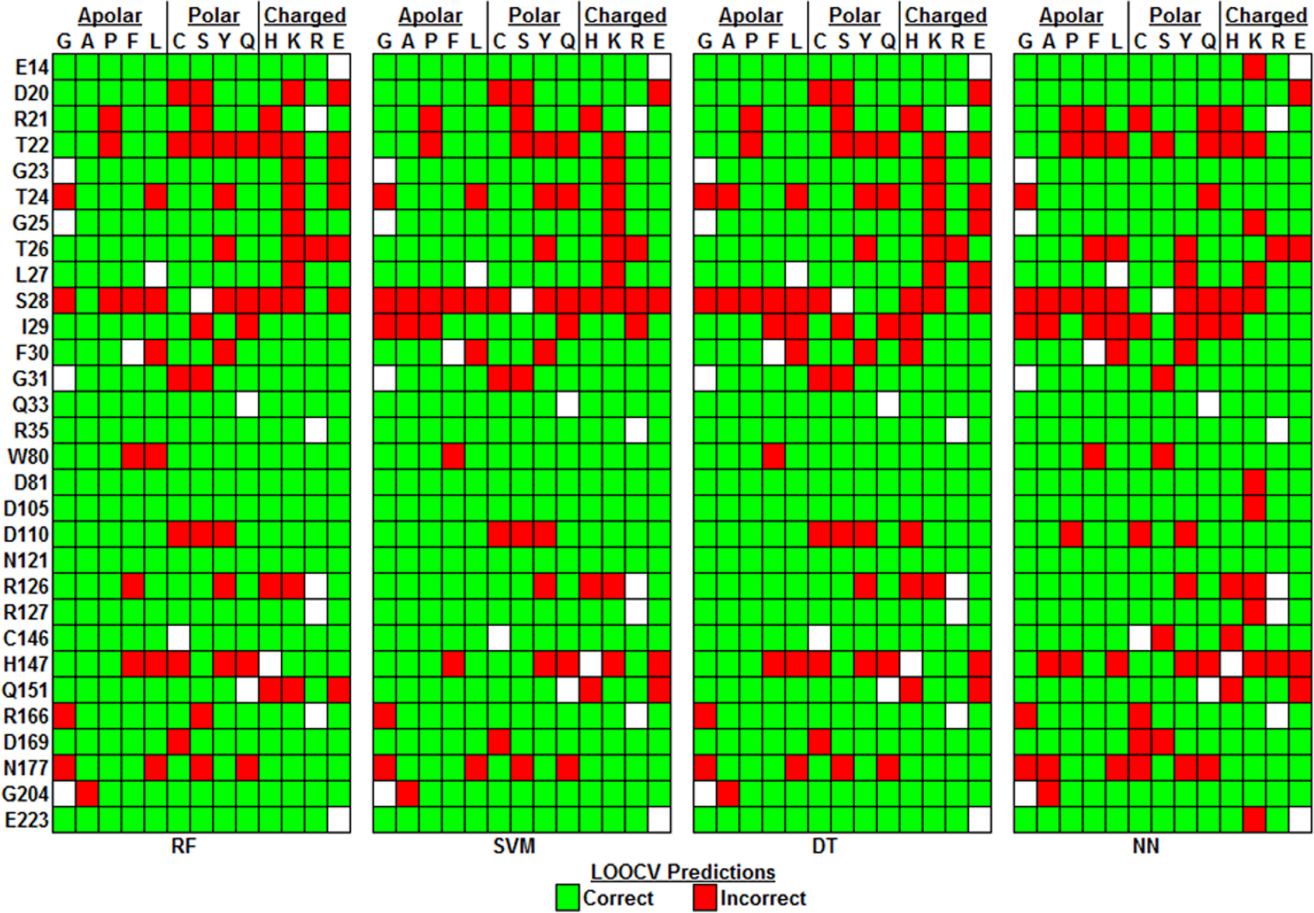
Model predictions. Visualization of *E. coli* thymidylate synthase (TS) variant-specific prediction results based on leave-one-out (LOOCV) testing.

Illustrated in Fig. 7 are the individual TS variant prediction results, obtained by LOOCV testing of the four supervised classification models, which were subsequently used for computing the summary performance data reported in Table 3. Collectively, 70% of the TS variants (259/372) were correctly predicted by all four methods, and an additional 11% (42/372) were misclassified only once; on the other hand, 10% of the variants (38/372) presented a challenge and were incorrectly predicted by every method. With respect to the individual TS sequence positions, all single residue substitutions at Q33, R35, and N121 were correctly predicted by all four methods. Nearly perfect predictions were also observed at E14, D81, D105, R127, and E223, with the NN algorithm causing a single misclassification at each position for the variant formed by introducing lysine (K) as the replacement residue. As discussed in the Introduction, these eight positions are among those that were experimentally determined to be highly substitutable, so the models were capable of accurately predicting variants for which activity was unaffected. At the other extreme, position S28 displayed the greatest number of variants (6 out of 12) that were incorrectly predicted by all four methods, followed by T22 with 4 out of 13 such misclassified variants; furthermore, fewer than half the variants at each of the positions T22, S28, I29, and H147 were correctly classified by more than two of the methods. Again referring to the Introduction, the latter residue position H147 was found to accept a limited number of substitutions, while the other three positions were determined to be highly sensitive to amino acid replacements. Consequently, the ability of models to correctly predict variants at these four positions presented a challenge.

The LOOCV predictions associated with each method were further examined by assessing the accuracy of TS variant subsets based on depth and secondary structure associated with the amino acid positions undergoing mutation, as well as by evaluating the performance of variant subsets according to the polarities of their native and replacement residues. Summaries of these data are presented in Tables 4 and 5, respectively, whereby each BAR and MCC accuracy measure represents the average value over all four methods, and % refers to the proportion of the 372 TS variants belonging to each category. Table 4 reveals that variants in helices were correctly classified more often than those in strands or coils, while predictions for mutations at surface and buried residues were substantially more accurate than those at undersurface positions. Moreover, substitutions of charged native residues were more accurately predicted than those of polar or hydrophobic/apolar native positions, as presented in Table 5 (column labeled All). Polar to charged and polar to polar residue replacements accounted for the top-most and third-highest misclassification rates, respectively, while representing a sizeable proportion of the TS variants at 12% and 22%, and these data are consistent with the reduced accuracy reported for undersurface positions. Conversely, variants incorporating apolar residues as replacements are correctly classified at a higher rate than those that use polar or charged amino acids as substitutions (Table 5, row labeled All). In particular, charged to apolar residue replacements displayed the highest accuracy rates.

**Table 4:** Mean LOOCV prediction performance based on depth and secondary structure.

	BAR	MCC	%
**Depth**			
Buried	0.83	0.67	50
Undersurface	0.60	0.21	20
Surface	0.91	0.79	30
**Secondary structure**			
Strand	0.78	0.57	46
Helix	0.88	0.76	21
Coil	0.82	0.63	33

**Table 5:** Mean LOOCV prediction performance based on side chain polarities of the native and new amino acids at the mutated position.

New/native	Polar			Apolar			Charged			All		
	BAR	MCC	%	BAR	MCC	%	BAR	MCC	%	BAR	MCC	%
Polar	0.75	0.50	22	0.80	0.60	16	0.73	0.46	12	0.76	0.52	50
Apolar	0.78	0.54	5	0.74	0.49	3	0.85	0.62	2	0.78	0.54	10
Charged	0.86	0.70	19	0.96	0.93	13	0.89	0.79	8	0.90	0.79	40
All	0.80	0.59	46	0.86	0.72	32	0.80	0.59	22	0.82	0.63	100

### Learning curves

**Figure 8:**
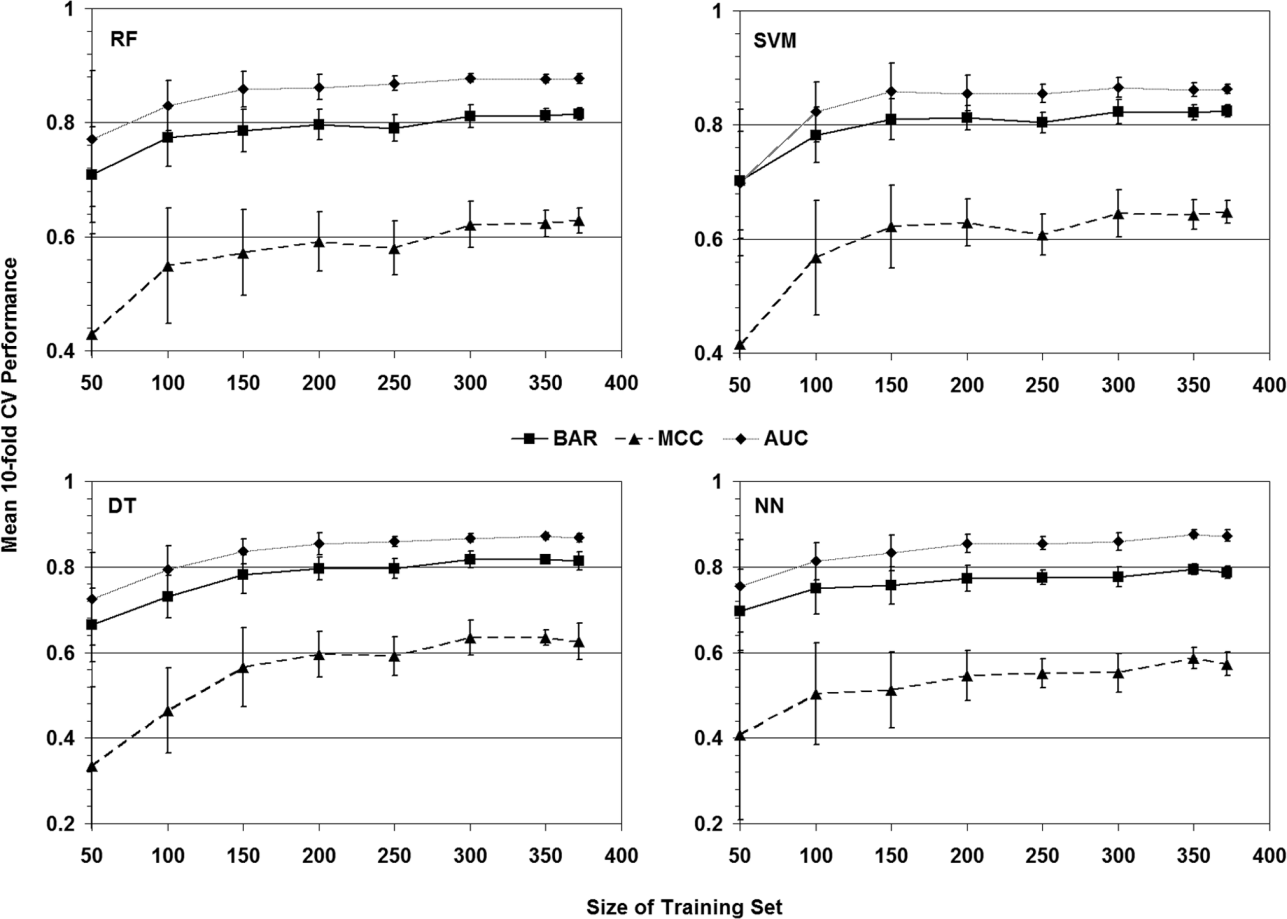
Learning curves. At each training set size increment and for each machine learning method, mean tenfold cross-validation (10-fold CV) performance measures were calculated for balanced accuracy rate (BAR), Matthew’s correlation coefficient (MCC), and area under the ROC curve (AUC).

Lastly, learning curves were generated as a way to visualize the effect of training set size on model performance. Using each machine learning method, tenfold cross-validation (10-fold CV) was applied to ten stratified random samples each consisting of 50 TS variants, whereby each set was selected from among all 372 TS variants, and mean BAR, MCC, and AUC values were calculated over all ten sets along with respective standard deviations. Subsequent iterations incremented the set sizes by 50 variants until sets of size 350 variants each were selected, and a final iteration consisted of running 10-fold CV testing ten times on the full set of 372 variants. The plots appear to plateau as the set size approaches 372 variants (Fig. 8), suggesting that optimal performance may have been achieved and that additional TS variant data may not necessarily improve accuracy.

### Concluding remarks

In this report, a knowledge-based four-body statistical potential energy function was used to empirically calculate a structural residue environment score for every amino acid position of the *E. coli* thymidylate synthase (TS) enzyme. An *in silico* mutagenesis procedure that relies on this energy function was implemented to characterize single residue TS variants in terms of a global structural perturbation score (i.e., the residual score), as well as local environmental perturbation (EP) scores at the mutated position and all structurally nearest-neighbor residues. When compared with available experimental data, these scores were shown to be effective at elucidating statistically significant TS structure–function relationships, distinguishing roles of TS residues, and training predictive models for classifying TS variant activity.

The available experimental dataset consisted of 372 single residue TS variants defined by introducing the same 12/13 amino acid substitutions at each of 30 TS positions, and each variant was determined to have either unaffected or detrimentally affected activity relative to the native enzyme. Despite such a restricted set of 201 unaffected and 171 affected TS variants, the overall average structural perturbation score (i.e., mean residual score) for the unaffected class of variants was near zero; however, the mean residual score for variants in the affected class was negative, reflecting a statistically significant difference between the mean residual scores of both classes and elucidating an inherent TS structure (i.e., mean residual score)–function (i.e., activity class) relationship.

More generally, residual scores were calculated for all TS variants (i.e., each of the 19 possible amino acid replacements of the native residue at every TS position) without regard to availability of experimental activity data, and a CMP (i.e., comprehensive mutational profile) score was calculated for each TS position by averaging the residual scores of all 19 variants associated with each position. Interestingly, a strong inverse correlation was observed between the (native) structural residue environment scores and the (variant) CMP scores over all TS positions, and a graphical display of this correlation reveals a clustering of TS positions based on native residue polarities. Also, substantial differences in these scores were observed between groups of TS residues annotated for known structural (buried, exposed) or functional (catalytic, interface) roles in the protein.

Finally, each TS variant in the experimental dataset was represented as a vector of features that included local EP scores at the mutated position and its six structurally nearest neighbors, specific type of residue replacement at the mutated position defining the variant, and additional sequence as well as structure based attributes. Combined with the known activity categories to which the 372 TS variants belong, this dataset was used to train and analyze predictive models of TS variant activity by implementing a variety of statistical machine learning algorithms. Cross-validation results suggest that the models are generally reliable and expected to perform well specifically with regards to predicting all currently unexplored TS variants (i.e., 7/8 amino acid replacements) at the 30 protein positions included in the training dataset. As more TS variant activities at additional positions become known, important goals with respect to this work will be to strengthen the aforementioned structure–function relationship and correlations, as well as to develop protein-wide predictive models.
